# The Risk of Suicide in the Post-COVID-19 Emergency Era: Psychological and Forensic Implications

**DOI:** 10.7759/cureus.49081

**Published:** 2023-11-19

**Authors:** Matteo Antonio Sacco, Saverio Gualtieri, Pietrantonio Ricci, Isabella Aquila

**Affiliations:** 1 Institute of Legal Medicine, Department of Medical and Surgical Sciences, Magna Græcia University of Catanzaro, Catanzaro, ITA

**Keywords:** coronavirus disease 2019, public health, covid 19, mental health, suicide, suicide prevention

## Abstract

The emergence of the coronavirus disease 2019 (COVID-19) pandemic brought numerous challenges, including the management of psychological and psychiatric disorders, leading to an increased risk of suicide. At the end of the COVID-19 emergency, we wonder what the impact of the pandemic has been, and still is, on the state of public mental health with respect to the phenomenon of suicide. Therefore, this review aims to explore the psychological and forensic aspects of suicide in the post-COVID-19 emergency era. The paper will delve into the various psychiatric disorders associated in the literature with COVID-19, the risk factors for suicide during the pandemic, and measures that can be taken to prevent suicide in the post-COVID-19 era. Additionally, the paper will look at how forensic experts investigate suicide cases due to COVID-19 and the legal implications of suicide due to the pandemic. The findings of this study will provide insights into the psychological and forensic aspects of suicide in the post-COVID era, and emphasize interventions and policy development to address this growing public health concern.

## Introduction and background

Exploring the global epidemiology of suicide

Suicide is a severe public health concern, as it is the fourth most common cause of death for people aged 15-29 years. In 2019, it is estimated that more than 700,000 people died by suicide [1]. A suicide attempt is an act in which a person attempts to take his own life but fails. In medicolegal literature, attempted suicide is considered an important factor for suicide [2]. Suicide attempts are over 30 times more frequent than actual suicides, with males being more at risk than females. According to research, the most common methods include hanging, poisoning using pesticides, and firearm usage [3,4]. Globally, depression is the primary cause of suicide deaths [4], while anxiety, personality, eating, trauma-related, and organic mental disorders significantly amplify the risk of suicide. A Swedish national cohort study has indicated that 12-19% of suicides were among depressed inpatients [5]. Nevertheless, the access to treatment and symptom severity affect the number of people who receive treatment for depression, with over 50% of depressed people in high-income countries and over 85% in low and middle-income countries remaining untreated for a year. Suicidal tendencies have been found to increase with the presence of physical or mental comorbidities, and even more so when hospitalized [3]. As such, it is imperative for mental health policies to address the global incidence of suicide and its risk factors, as well as develop preventive strategies [4].

The impact of pandemics on suicidal risk throughout history

The literature suggests that during a pandemic there is a substantial increase in psychiatric pathologies and mental health disorders, as was observed in the past during epidemics like the Spanish Flu and severe acute respiratory syndrome (ARDS). Various studies have tried to understand the impact of historical pandemics on suicide rates. A study showed that around 50% of hospitalized patients remained anxious after the severe acute respiratory syndrome (SARS) epidemic in Hong Kong in 2003, and social disengagement was found to have played a role in the increased suicide rate [6,7]. Wasserman's research evidenced certain indicators such as alcohol prohibition, publicly reported suicide stories, and unemployment [8-10]. The Russian Influenza between 1889 and 1893 was associated with an increase in the suicide rate in different regions of the United Kingdom [11]. Similarly, a marked rise in the suicide rate was observed in the United States during the Great Influenza Epidemic between 1910 and 1920 [8]. During this epidemic, the suicide rate peaked at 8.5/100,000, which was the highest on record. Furthermore, the suicide rate increased by 25% between 1889 and 1893, as determined by coroners' verdicts [8,12]. The mortality rate during the Spanish Flu has been associated with an increase in suicide percentages [9,10]. Additionally, there is evidence of increased suicide reports among older people during SARS and the year following the epidemic [12]. This may be due to social disconnectedness, fear of infection, and concern about burdening others. Similarly, numerous studies reported associations between coronavirus disease 2019 (COVID-19) distress and suicidal ideation [13,14]. 

## Review

Risk factors of suicide due to COVID-19 pandemic

It is clear from numerous studies that the COVID-19 pandemic has caused an escalation in suicide risk factors [8,9]. It is believed that this increase is due to a combination of pandemic-specific factors such as social isolation and economic hardship, as well as pre-existing factors such as social crises in certain regions and inadequate mental health services. The COVID-19 pandemic has further exacerbated the risk factors associated with suicidal behavior, including social distancing leading to strained social and familial relationships, more pronounced feelings of loneliness, inactivity, and boredom, and limited access to healthcare services [8].

The impact of the COVID-19 outbreak on people's mental health has been immense, causing widespread concern for the physical wellbeing of themselves and their loved ones [15]. The United States has seen a significant rise in calls to suicide prevention hotlines, and several cases of COVID-19-related suicides have been reported in various countries [16,17]. This phenomenon has affected a large number of individuals, including healthcare workers who have been in close contact with COVID-19 patients and have shown an increased risk of suicide [18]. According to a study in China, 96.2% of COVID-19 patients who recovered experienced significant posttraumatic stress symptoms [19]. These symptoms can be attributed to the traumatic experiences of being diagnosed with the illness, the fear of infecting others, the various symptoms associated with the illness, hospitalization, admission to intensive care units, and loss of income. Additionally, some individuals have reported experiencing prolonged physical symptoms, such as pain and loss of smell and taste, which can increase the risk of suicide. Yom-Tov et al. have reported long-term effects on patient mental health associated with COVID-19-induced anosmia and ageusia [20].

COVID-19 pandemic and psychiatric disorders

According to studies, mental health problems like depression have been more prevalent during the COVID-19 pandemic than during previous epidemics such as SARS [21]. This is due to the virus being more infectious and spreading more rapidly, leading to an increase in mental symptoms across all populations [22]. The impact of the pandemic on mental health has varied from country to country and across different groups, including the general public, health workers, university students, older adults, infected patients, survivors, and pregnant women. Depression, anxiety, and insomnia have all increased as a result of the pandemic [23]. However, infected patients have experienced a decrease in mental symptoms, possibly due to their recovery. Poor mental health during the pandemic has been linked to factors such as the risk of infection, the presence of COVID-19-like symptoms, mask shortage, and unclear mask reuse guidelines. Furthermore, the lockdowns and home confinement measures implemented during the pandemic may have worsened existing mental health conditions. It is important to note that the mental health response to the pandemic may vary significantly.

The literature has exhibited a connection between psychiatric disorders and the pandemic, with a wide range of disorders being identified. For example, the implementation of lockdowns and social distancing has resulted in a sense of isolation and disconnection for many people, with vulnerable and disadvantaged populations experiencing a higher prevalence of mental health issues [24]. Moreover, individuals with pre-existing psychiatric disorders have been impacted more significantly by the pandemic, mainly due to interruptions in their mental health services and the increased prevalence of mental health issues among those with risk factors [25]. The literature has also reported that the effects of the pandemic on mental health have been compounded by various factors such as fear of contagion, economic difficulties, and vaccine hesitancy [26], all of which can lead to stress-related disorders [4].

COVID-19 and forensic implications

The COVID-19 pandemic has had a significant impact on the field of forensic investigations, requiring professionals to adapt quickly to the changes brought on by the pandemic. Guidelines have been established for autopsy procedures during the pandemic, which include recommendations for ventilation devices, air exchange, and the use of high-efficiency particulate aerosol (HEPA) filters. The pandemic has also affected crime scene investigations, with a number of safety protocols being implemented to prevent the spread of COVID-19 [27]. These protocols include proper hygiene procedures like frequent hand washing and the use of hand sanitizer, as well as the use of personal protective equipment (PPE) such as masks, gloves, and gowns. Investigators are also advised to maintain social distancing guidelines during investigations and avoid physical contact with surfaces as much as possible. To reduce the risk of infection, it is important to minimize the number of personnel present during investigations. It is recommended that two pairs of gloves or specialized protective gloves be worn during crime scene investigations [28]. Due to the COVID-19 pandemic and subsequent lockdown measures, there has been a noticeable decrease in both crime scene investigations and autopsies. These decisions were made to limit the spread of the virus, but have affected the accurate analysis of suicides, particularly in relation to their correlation with COVID-19.

Forensic investigations of suicide cases due to COVID-19

In order to investigate cases of suicide related to COVID-19, experts in the field of forensic literature have employed a combination of psychiatric, medico-legal, and psychological examinations. One such method is the psychological autopsy, which involves interviewing relatives and caregivers of the deceased to gain insight into the individual's mental state before their passing. This technique is highly regarded for its ability to identify the causes and triggers of suicide, making it an invaluable tool in suicide research. It is a direct and effective way of examining the relationship between antecedents and suicide, and is becoming increasingly important in this field. The psychological autopsy involves gathering information from various sources, such as family members and healthcare personnel, as well as records. It has been utilized to study the link between the COVID-19 pandemic and suicide, and has been applied to numerous forensic cases involving severe acute respiratory syndrome coronavirus 2 (SARS‑CoV‑2) infection [29]. The investigation of these cases involves analyzing medical records and assessing the mental state of the deceased. Performing an autopsy is crucial to ensure accurate information is obtained, specifically to rule out any injuries inflicted by external parties and to examine any damage in greater detail. Research conducted on suicides related to COVID-19 has shown that the motivations behind these acts are multifaceted, encompassing both social and economic consequences of the pandemic, as well as the SARS-CoV-2 infection itself. Individuals without prior psychiatric conditions also exhibited mental deterioration, particularly in the form of depressive and anxious symptoms. Forensic studies have demonstrated that the psychological autopsy represents a valuable tool for investigating COVID-19-related suicides. However, it is important to acknowledge limitations, such as the potential for interview bias and variations in the quality and quantity of information provided by subjects [30-34].

To properly investigate suicides that have occurred during the pandemic, it is crucial to ensure the safety, confidentiality, and integrity of everyone involved. This investigation must also take into account all potential risk factors that could have contributed to suicide related to COVID-19. The psychological autopsy method, as well as autopsies, should be used to support any information obtained from the scene investigation. It's important to note that emotional distress is a significant burden for both interviewers and respondents during such investigations. The pandemic has intensified depressive and anxiety symptoms, which is why governments must continue to monitor and report suicide mortality in real time for forensic purposes. Investigators must also pay attention to the risk factors of suicide, considering the possibility that it could be directly or indirectly related to COVID-19. It's crucial to understand that the pandemic has been linked to individual cases of suicide. The COVID-19 pandemic has brought psychosocial stressors to the forefront, with suicide rates being particularly affected [35]. SARS-CoV-2 has been known to cause psycho-emotional consequences that may lead to suicide, regardless of clinical severity. In light of this, mental health professionals must remain vigilant in suicide prevention efforts for individuals infected with SARS-CoV-2 even after the emergency has ended. It is crucial that patients infected with SARS-CoV-2 who possess suicidal risk factors receive thorough surveillance [9].

Suicidal trends during and after COVID-19 lockdown

Regarding the number of suicides that have occurred, recent literature suggests that the trend, i.e. the global number of suicides that occurred during the pandemic and in particular during the lockdown phases in 2020, has not substantially increased in adults in various countries around the world [36]. However, several authors have highlighted how, beyond the overall number of suicides, the presence of various phenomena worthy of further investigation has been noted. In Spain, an increase in suicide attempts among adolescents during the COVID-19 pandemic was highlighted during the lockdown period [37]. In Saudi Arabia, a statistically significant increase in inpatient psychiatric services utilization was highlighted during and after the lockdown [38]. In Denmark, an increase in suicide attempts was observed among men in the post-COVID-19 lockdown [39]. In France, although there was no evidence of an increase in suicide mortality during the pandemic, an increase in suicides was observed after the third lockdown from mid-2021 to March 2022 in 18-24-year-olds and men aged 65-84 years [40]. In Italy, it has been shown that adolescents in the second lockdown period more frequently showed self-harming behavior, suicidal ideation, and suicide attempts [41]. In Serbia, an increase in suicidal ideation in a group of 104 psychiatric patients was observed in 2020, compared to the pre-COVID-19 phase [42]. In Salamanca, an increase in suicide attempts was observed after the declaration of alarm due to COVID-19 [43]. The data suggests that geographical variations can be found in the phenomenon. It is important to note that although a reduction in the global number of suicides has been observed in some countries, the same cannot be said with respect to the phenomena of suicidal ideation and suicide attempts, especially in more fragile groups such as adolescents.

What measures can be taken to prevent suicide in the post-COVID-19 era?

Preventing suicide in the post-COVID-19 era requires a variety of measures. Community-based training programs should be implemented to identify those most in need and provide timely intervention on a regular basis. Virtual platforms can also be utilized to screen for early signs of risk. Early detection of suicidal behavior is crucial, and interventions must be provided in a timely manner. Media outlets must avoid instilling fear and hopelessness in the community. To mitigate the pandemic's impact on the economy, payment policies and economic supports must be revised. Mental health and psychological well-being programs should be designed and provided for those at risk, including awareness programs and the promotion of social connectedness. Communication channels should be made available and access to interventions for those at risk must be increased.

## Conclusions

The COVID-19 pandemic has had a profound impact on the mental well-being of people on a global scale, resulting in increased risk factors for suicide and psychiatric disorders. Social isolation, the fear of contracting the virus, and disruptions to mental health services have all contributed to a potential rise in suicidal behavior during the pandemic. Moreover, limited access to hospitals and healthcare providers has led to more severe suicide attempts. The pandemic has also aggravated pre-existing psychiatric disorders. Although there are still discussions about the epidemiological trend during the pandemic, the literature nevertheless demonstrates how important risk factors for public mental health have emerged. 

To prevent suicide in the post-COVID-19 era, mental health awareness programs and initiatives to promote social connectedness are necessary. Healthcare providers should develop a systematic suicide screening process to identify vulnerable individuals and increase suicide risk screening. It is essential to continue monitoring the correlation between COVID-19 and suicide for epidemiological and forensic purposes. In order to better understand the enduring impact of the pandemic on mental health, forthcoming studies should concentrate on long-term effects, particularly the possibility of amplified suicide risk in the aftermath. It is imperative that mental health resources and support remain a priority even after the COVID-19 pandemic to combat and address the growing prevalence of psychiatric disorders and suicide.
